# Supplementary material for: A Novel Method to Screen Strong Constitutive Promoters in Escherichia coli and Serratia marcescens for Industrial Applications
Source: Biology (Basel). 2022 Dec 30;12(1):71. doi: 10.3390/biology12010071 (PMC9855843; doi:10.3390/biology12010071)
Supplement: Supplementary file 1 [file biology-12-00071-s001.zip › biology-2069770-supplementary.pdf]

A Novel Method to Screen Strong Constitutive Promoters in *Escherichia coli* and *Serratia marcescens* for Industrial Applications

Xuewei Pan[1,2], Mi Tang[1], Jiajia You[1], Yanan Hao[1], Xian Zhang[1], Taowei Yang[1*], Zhiming Rao[1]

1. Key Laboratory of Industrial Biotechnology of the Ministry of Education, Laboratory of Applied Microorganisms and Metabolic Engineering, School of Biotechnology, Jiangnan University, Wuxi 214122, China

2. School of Food Science and Technology, Jiangnan University, Wuxi 214122, China

* Correspondence: yangtw@jiangnan.edu.cn; Tel.: +86-510-85916881

Table S1. Strains and plasmids used in this study

Strains/Plasmids	Characteristics	Source
**Strains**		
Escherichia coli JM109	Host for cloning	Lab stock
E. coli MG1655	Wild-type strain	Lab stock
Corynebacterium glutamicum ATCC13032	Wild-type strain	Lab stock
Bacillus subtilis 168	Wild-type strain	Lab stock
Serratia marcescens JNB5-1	Wild-type strain	Lab stock
Val01	Strain obtained by ARTP mutagenesis derived from *E. coli* W3110	This study
Val02	Val01 derivative with expression of *ilvCDE*, controlled by P_{BS76}-50 promoter	This study
Val03	Val01 derivative with expression of *ilvCDE*, controlled by P_{BS76}-75 promoter	This study
Val04	Val01 derivative with expression of *ilvCDE*, controlled by P_{BS76}-85 promoter	This study
Val05	Val01 derivative with expression of *ilvCDE*, controlled by P_{BS76}-100 promoter	This study

SM01	JNB5-1 derivative with expression of *pigFN*, controlled by P_{pig} promoter	This study
SM02	JNB5-1 derivative with expression of *pigFN*, controlled by P_{RpIJ} promoter	This study
SM03	JNB5-1 derivative with expression of *pigFN*, controlled by P_{SM} promoter	This study
**Plasmids**		
pUC19	Lac promoter, pBR322 origin, Amp^R	Lab stock
pUC19-P_{BBa_23118}-*gfp*	pUC19 derivative with *gfp* driven by constitutive promoter P_{BBa_J23118}	This study
pUC19-P_{EC}-*gfp*	pUC19 derivative with *gfp* driven by promoter P_{EC} screened from *E. coli* MG1655	This study
pUC19-P_{BS}-*gfp*	pUC19 derivative with *gfp* driven by promoter P_{BS} screened from *B. subtilis* 168	This study
pUC19-P_{CG}-*gfp*	pUC19 derivative with *gfp* driven by promoter P_{CG} screened from *C. glutamicum* ATCC13032	This study
pUC19-P_{BS76}-*gfp*	pUC19 derivative with *gfp* driven by promoter P_{BS76} truncated from promoter P_{BS}	This study
pUC19-$P_{BS76\text{-}variant}$-*gfp*	pUC19 derivative with *gfp* driven by promoter $P_{BS76\text{-}variant}$ optimized based on promoter P_{BS76}	This study
pUC19-P1-*gfp*	pUC19 derivative with *gfp* driven by truncated promoter P1 derived from promoter P_{BS}	This study
pUC19-P2-*gfp*	pUC19 derivative with *gfp* driven by truncated promoter P2 derived from promoter P_{BS}	This study

pUC19-P3-*gfp*	pUC19 derivative with *gfp* driven by truncated promoter P3 derived from promoter P_{BS}	This study
pUC19-P4-*gfp*	pUC19 derivative with *gfp* driven by truncated promoter P4 derivedfrom promoter P_{BS}	This study
pUC19-P5-*gfp*	pUC19 derivative with *gfp* driven by truncated promoter P5 derived from promoter P_{BS}	This study
pUC19-P6-*gfp*	pUC19 derivative with *gfp* driven by truncated promoter P6 derived from promoter P_{BS}	This study
pUC19-P7-*gfp*	pUC19 derivative with *gfp* driven by truncated promoter P7 derived from promoter P_{BS}	This study
pUC19-P8-*gfp*	pUC19 derivative with *gfp* driven by truncated promoter P8 derived from promoter P_{BS}	This study
pUC19-P9-*gfp*	pUC19 derivative with *gfp* driven by truncated promoter P9 derived from promoter P_{BS}	This study
pUC19-P10-*gfp*	pUC19 derivative with *gfp* driven by truncated promoter P10 derived from promoter P_{BS}	This study
pUC19-P11-*gfp*	pUC19 derivative with *gfp* driven by truncated promoter P11 derived from promoter P_{BS}	This study
pUC19-P12-*gfp*	pUC19 derivative with *gfp* driven by truncated promoter P12 derived from promoter P_{BS}	This study
pTrc99a	Expression vector, trc promoter, Amp^R	Lab stock
pTrc99a-P_{BS}-50-*ilvCDE*	Expressing *ilvCDE* via pTrc99a under the control of promoter P_{BS}-50	This study
pTrc99a-P_{BS}-75-*ilvCDE*	Expressing *ilvCDE* via pTrc99a under the control of promoter P_{BS}-75	This study

pTrc99a-P_{BS}-85-*ilvCDE*	Expressing *ilvCDE* via pTrc99a under the control of promoter P_{BS}-85	This study
pTrc99a-P_{BS}-100-*ilvCDE*	Expressing *ilvCDE* via pTrc99a under the control of promoter P_{BS}-100	This study
pUCP18	Broad-host-range shuttle vector, Amp^R	Lab stock
pUCP18-P_{SM}-*gfp*	pUCP18 derivative with *gfp* driven by promoter P_{SM} screened from *S. marcescens* JNB5-1	This study
pUCP18-P_{pig}-*gfp*	pUCP18 derivative with *gfp* driven by the native promoter P_{pig}	This study
pUCP18-P_{pig}-*pigFN*	pUCP18 derivative with *pigFN* driven by native promoter P_{pig}	This study
pUCP18-P_{RpIJ}-*pigFN*	pUCP18 derivative with *pigFN* driven by constitutive promoter P_{rpIJ}	This study
pUCP18-P_{SM}-*pigFN*	pUCP18 derivative with *pigFN* driven by promoter P_{SM} screened from *S. marcescens* JNB5-1	This study

Table S2. Primers used in this study

**Primers**	**Sequences (5'-3')**	**Function**
p19-GFP-F1	AGTACCATGATTACGCCAAGCTTG	Primers used to construction of the plasmid pUC19-P_{BBa_23118}-*gfp*
p19-GFP-R1	TCACAATTCCACACATTATACGAGCCGGATGATTAATTGTCAAGCCTGGGGTGCCTAATGAGT	
p19-GFP-F2	TAATGTGTGGAATTGTGAGCGGATAACAATTTCACACAGGAAACAGCTATGAGTAAAGGAGAAGAACTTTTCACTGGAGT	
p19-GFP-R2	TGGCGTAATCATGGTCATCTATTTGTATAGTTCATCCATGCCATGTGTAATCC	
promoter-BS-F1(116)	TAAAGAGGAGAAAGGTACCCGCATAATAAAGGAAAAAGCAGGCGCATG	Primers used to identify the core region of the promoter P_{BS}

promoter-BS-F1(96)	TAAAGAGGAGAAAGGTACCCGGCGCATGG ATATAAGGCGC
promoter-BS-F1(76)	TAAAGAGGAGAAAGGTACCCCTGCTTTTTT ATTGTTGAAAGCGCTTTATTTTTCCC
promoter-BS-F1(56)	TAAAGAGGAGAAAGGTACCCGCGCTTTATT TTTCCCCTACAATAGATGAAAACG
promoter-BS-F1(36)	TAAAGAGGAGAAAGGTACCCAATAGATGA AAACGGCGTGTAAGGGAG
promoter-BS-F1(16)	TAAAGAGGAGAAAGGTACCCAAGGGAGGA GCGATCCATGAGTAAA
promoter-BSTY-R1	CTATTTGTATAGTTCATCCATGCCATGTGTA ATCC

promoter-BSTY-F2	GGATGAACTATACAAATAGCCGGGTACCGA GCTCGAATTCA
promoter-BSTY-R2	GGGTACCTTTCTCCTCTTTAATGAATTCGC
promoter-BS76TY-F1	ATGAGTAAAGGAGAAGAACTTTTCACTGGA GT
promoter-BS76TY-R1	CTATTTGTATAGTTCATCCATGCCATGTGTA ATCC
promoter-BS76TY-F2	GGATGAACTATACAAATAGCCGGGTACCGA GCTCGAATTCA
promoter-BS76-R2(56)	AGTTCTTCTCCTTTACTCATGCCGTTTTCATC TATTGTAGGGGAAAAATAAAG
promoter-BS76-R2(36)	AGTTCTTCTCCTTTACTCATGGGAAAAATAA AGCGCTTTCAACAATAAAAAAGC

promoter-BS76-R2(16)	AGTTCTTCTCCTTTACTCATAACAATAAAAAAGCAGGGGTACCTTTCTCC	
p19-BS(76)10and1NN-F1	CTTTATTTTTCCCCTACAATNNNNNNNNNACGGCGTGTAAGGGAGGAG	Primers used to construction of a gradient promoter library *via* modifying promoter P_{BS76}
p19-BS(76)10NN-F1	AAAGCGCTTTATTTTTCCCCNNNNNNAGATGAAAACGGCGTGTAAGGGAG	
p19-BS(76)35and10NN-F1	CTGCTTTTTTATTGTTGAAANNNNNNNNNNNNNNNNNTACAATAGATGAAAACGGCGTGTAAGGGAG	
p19-BS(76)35NN-F1	CCAGGACTGCTTTTTTATTGNNNNNNGCGCTTTATTTTTCCCCTACAATAGATGAAAACG	
p19-BS(76)10and1NN-R2	ATTGTAGGGGAAAAATAAAGCGCTTTCAACA	

p19-BS(76)10NN-R2	GGGGAAAAATAAAGCGCTTTCAACAATAAA AAAGC	
p19-BS(76)35and10NN-R2	TTTCAACAATAAAAAAGCAGGCCTGGGG	
p19-BS(76)35NN-R2	CAATAAAAAAGCAGGCCTGGGGTG	
p19-BS(76)TY-R1	CTATTTGTATAGTTCATCCATGCCATGTGTA ATCCC	
p19-BS(76)TY-F2	TGGATGAACTATACAAATAGCCGGGTACC	
promoter-BS-F1(76)	CTGCTTTTTTATTGTTGAAAGCGCTTTATTTT TCCC	Primers used to construction of the plasmid pUC19-P_{BS76}-*gfp*
promoter-BS-R1(76)	CTATTTGTATAGTTCATCCATGCCATGTGTA ATCCC	
promoter-BS-F2(76）	TGGATGAACTATACAAATAGCCGGGTACC	

promoter-BS-R2(76)	TTTCAACAATAAAAAAGCAGGCCTGGGGTG<br>CCTAATGAGT	
18-Ppig-pigFN-F1	AAAACGACGGCCAGTGCCAAGCTTTTTTTCC<br>TCCGGAATGCTCCTGC	Primers used to construction of the plasmid pUCP18-P_{pig}-*pigFN*
18-Ppig-pigFN-R1	TCTTGCTTGGTTAAAGGCATTGGGTTGAGAG<br>ATTAAATTAGCTAATATTTCTAGTTTGGAGG	
18-Ppig-pigFN-F2	TAATTTAATCTCTCAACCCAATGCCTTTAAC<br>CAAGCAAGATGCC	
18-Ppig-pigFN-R2	AAAGCAATCCATACATTCATTTATTTTTCGC<br>CGACGATCAGGGT	
18-Ppig-pigFN-F3	TGATCGTCGGCGAAAAATAAATGAATGTAT<br>GGATTGCTTTGGCCGT	

18-Ppig-pigFN-R3	TTCGAGCTCGGTACCCGGGGATCCTTACAG CACGAAAGGAATGAAACACTTAACCT	
18-PrplJ-pigFN-F1	AAAACGACGGCCAGTGCCAAGCTTTCGCAC TTGCGATTATCGCTTTG	Primers used to construction of the plasmid pUCP18-P_{rplJ}-*pigFN*
18-PrplJ-pigFN-R1	TCTTGCTTGGTTAAAGGCATTAGCTTTTTGC TCCTGGATTAGCCG	
18-PrplJ-pigFN-F2	AATCCAGGAGCAAAAAGCTAATGCCTTTAA CCAAGCAAGATGCC	
18-PrplJ-pigFN-R2	AAAGCAATCCATACATTCATTTATTTTTCGC CGACGATCAGGG	
18-PrplJ-pigFN-F3	TGATCGTCGGCGAAAAATAAATGAATGTAT GGATTGCTTTGGCCG	

18-PrplJ-pigFN-R3	TTCGAGCTCGGTACCCGGGGATCCTTACAG CACGAAAGGAATGAAACACTTAACC	
18-PSM-pigFN-F1	AAAACGACGGCCAGTGCCAAGCTTGCCTGC CTTCCGTTCGTC	Primers used to construction of the plasmid pUCP18-P$_{SM}$-*pigFN*
18-PSM-pigFN-R1	TCTTGCTTGGTTAAAGGCATGAGACCAGAG CTCCAATTATTTATAAACGTAAATAATTACT C	
18-PSM-pigFN-F2	ATAATTGGAGCTCTGGTCTCATGCCTTTAAC CAAGCAAGATGCC	
18-PSM-pigFN-R2	AAAGCAATCCATACATTCATTTATTTTTCGC CGACGATCAGGG	
18-PSM-pigFN-F3	TGATCGTCGGCGAAAAATAAATGAATGTAT GGATTGCTTTGGCCG	

18-PSM-pigFN-R3	TTCGAGCTCGGTACCCGGGGATCCTTACAG CACGAAAGGAATGAAACACTTAACC	
18-Ppig-gfp-F1	AAAACGACGGCCAGTGCCAAGCTTTTTTTCC TCCGGAATGCTCCTGC	Primers used to construction of the plasmid pUCP18-P$_{pig}$-*gfp*
18-Ppig-gfp-R1	AGTTCTTCTCCTTTACTCATTGGGTTGAGAG ATTAAATTAGCTAATATTTCTAGTTTGGAGG	
18-Ppig-gfp-F2	TAATTTAATCTCTCAACCCAATGAGTAAAG GAGAAGAACTTTTCACTGGAGT	
18-Ppig-gfp-R2	TTCGAGCTCGGTACCCGGGGATCCCTATTTG TATAGTTCATCCATGCCATGTGTAATCCC	
18-PrplJ-gfp-F1	AAAACGACGGCCAGTGCCAAGCTTTCGCAC TTGCGATTATCGCTTTG	Primers used to construction of the plasmid pUCP18-P$_{rplJ}$-*gfp*

18-PrplJ-gfp-R1	AGTTCTTCTCCTTTACTCATTAGCTTTTTGCT<br>CCTGGATTAGCCG	
18-PrplJ-gfp-F2	AATCCAGGAGCAAAAAGCTAATGAGTAAAG<br>GAGAAGAACTTTTCACTGGAGT	
18-PrplJ-gfp-R2	TTCGAGCTCGGTACCCGGGGATCCCTATTTG<br>TATAGTTCATCCATGCCATGTGTAATCCC	
18-PSM-gfp-F1	AAAACGACGGCCAGTGCCAAGCTTGCCTGC<br>CTTCCGTTCGTC	Primers used to construction of the plasmid pUCP18-P_{SM}-*gfp*
18-PSM-gfp-R1	AGTTCTTCTCCTTTACTCATGAGACCAGAGC<br>TCCAATTATTTATAAACGTAAATAATTACTC	
18-PSM-gfp-F2	ATAATTGGAGCTCTGGTCTCATGAGTAAAG<br>GAGAAGAACTTTTCACTGGAGT	

18-PSM-gfp-R2	TTCGAGCTCGGTACCCGGGGATCCCTATTTG TATAGTTCATCCATGCCATGTGTAATCCC	
99a-BS50-ilvCDE-F1	AATCGAAACTGGGGGGTTAAGCTGTTTTGG CGGATGAGAGAAG	Primers used to construction of the plasmid pTrc99a-P$_{BS50}$-*ilvCDE*
99a-BS50-ilvCDE-R1	AGGGGAAAAATAAAGCGCCTTGATCAATAA AAAAGCAGGCTCATTTCAGAATATTTGCCA GAACCG	
99a-BS50-ilvCDE-F2	AGGCGCTTTATTTTTCCCCTACAATAGATGA AAACGGCGTGTAAGGGAGGAGCGATCCATG GCTAACTACTTCAATACACTGAATCTGC	
99a-BS50-ilvCDE-R2	TCAGCTTTCTTCGTGGTCATTTAACCCGCAA CAGCAATACGTT	

99a-BS50-ilvCDE-F3	GTATTGCTGTTGCGGGTTAAATGACCACGAAGAAAGCTGATTACATTT	
99a-BS50-ilvCDE-R3	TTAACCCCCCAGTTTCGATTTATCGC	
99a-BS75-ilvCDE-F1	GCTGTTTTGGCGGATGAGAGAAG	Primers used to construction of the plasmid pTrc99a-P_{BS75}-*ilvCDE*
99a-BS75-ilvCDE-R1	TAGGGGAAAAATAAAGCGCTTTCAACAATAAAAAAGCAGGCTCATTTCAGAATATTTGCCAGAACCG	
99a-BS75-ilvCDE-F2	AGCGCTTTATTTTTCCCCTACAATAACGTGCCACGGCGTGTAAGGGAGGAGCGATCCATGGCTAACTACTTCAATACACTGAATCTGC	
99a-BS75-ilvCDE-R2	CAGCTTTCTTCGTGGTCATTTAACCCGCAACAGCAATACGTT	

99a-BS75-ilvCDE-F3	CGTATTGCTGTTGCGGGTTAAATGACCACG AAGAAAGCTGATTACATTT	
99a-BS75-ilvCDE-R3	CTCTCATCCGCCAAAACAGCTTAACCCCCC AGTTTCGATTTATCGC	
99a-BS85-ilvCDE-F1	AGCTGTTTTGGCGGATGAGAGAAG	Primers used to construction of the plasmid pTrc99a-P_{BS85}-*ilvCDE*
99a-BS85-ilvCDE-R1	TGTAGGGGAAAAATAAAGCGCCTTGATCAA TAAAAAAGCAGGCTCATTTCAGAATATTTG CCAGAACCG	
99a-BS85-ilvCDE-F2	CGCTTTATTTTTCCCCTACAATAGATGAAAA CGGCGTGTAAGGGAGGAGCGATCCATGGCT AACTACTTCAATACACTGAATCTGC	
99a-BS85-ilvCDE-R2	AATCAGCTTTCTTCGTGGTCATTTAACCCGC AACAGCAATACGTT	

99a-BS85-ilvCDE-F3	ATTGCTGTTGCGGGTTAAATGACCACGAAG AAAGCTGATTACATTT	
99a-BS85-ilvCDE-R3	TCTCATCCGCCAAAACAGCTTAACCCCCCA GTTTCGATTTATCGC	
99a-BS100-ilvCDE-F1	TAAGCTGTTTTGGCGGATGAGAGAAG	Primers used to construction of the plasmid pTrc99a-P_{BS100}-*ilvCDE*
99a-BS100-ilvCDE-R1	TTGTAGGGGAAAAATAAAGCGCTTTCAACA ATAAAAAAGCAGGCTCATTTCAGAATATTT GCCAGAACCG	
99a-BS100-ilvCDE-F2	GCTTTATTTTTCCCCTACAATAGATGAAAAC GGCGTGTAAGGGAGGAGCGATCCATGGCTA ACTACTTCAATACACTGAATCTGC	
99a-BS100-ilvCDE-R2	TAATCAGCTTTCTTCGTGGTCATTTAACCCG CAACAGCAATACGTT	

99a-BS100-ilvCDE-F3	TTGCTGTTGCGGGTTAAATGACCACGAAGA AAGCTGATTACATTT
99a-BS100-ilvCDE-R3	TCATCCGCCAAAACAGCTTAACCCCCCAGT TTCGATTTATCGC
